# Incidence and outcomes for colonization of multidrug-resistance organisms (MDROs) among patients undergoing elective orthopedic surgery

**DOI:** 10.1017/ash.2026.10354

**Published:** 2026-04-16

**Authors:** Sirikwun Umpunthongsiri, Anucha Apisarnthanarak, Potjanee Srimanote, Thana Khawcharoenporn, Chayanin Aungthong, Narissara Mungkornkaew, Pansachee Damronglerd, Sasinuch Rutjanawech, Nuntra Suwantarat

**Affiliations:** 1 Division of Infectious Disease, Department of Medicine, Faculty of Medicine, Thammasat University, Thailand; 2 Faculty of Allied Health Science, Thammasat University, Thailand; 3 Department of Orthopedic, Faculty of Medicine, Thammasat University, Thailand; 4 Thammasat University Hospital, Thailand; 5 Chulabhorn International College of Medicine (CICM), Thammasat University, Thailand

## Abstract

**Objective:**

To evaluate the incidence, risk factors, and outcomes associated with multidrug-resistant organisms (MDROs) colonization in patients undergoing elective orthopedic surgery at the Thammasat University Hospital.

**Methods:**

We conducted a prospective MDROs surveillance screening (swabs from the nose, throat, groin, and rectum) in patients undergoing orthopedic surgery. MDROs were defined as extended-spectrum β-lactamase (ESBL)-producing Gram-negative bacteria (GNB), carbapenem-resistant *Enterobacterales*, methicillin-resistant *Staphylococcus aureus*, and vancomycin-resistant enterococci. The incidence of MDROs colonization, risk factors, and outcomes including surgical site infections (SSIs) were assessed. Postoperative SSIs were compared between patients with and without MDROs colonization.

**Results:**

Of 384 swabs tested from 96 patients, ESBL-producing *Escherichia coli* was identified in 38 isolates (31 rectal swabs and 7 groin swabs) from 31 patients (32.3%). Only one patient had a history of admission within the previous year. Majority of procedures involved prosthetic implantation (77.1%) including total knee arthroplasty (30.2%). Seven patients (7.3%) developed SSIs without microbiological confirmation. The incidence of SSIs was higher among patients with ESBL-producing *E. coli* colonization compared to patients without colonization (6/31, 19.4% vs. 1/65, 1.5%; *P* = .004; odds ratio, 15.36; 95% CI 1.7–356.3). From the multivariate logistic regression analysis, preoperative ESBL-producing *E. coli* colonization was associated with SSIs (*P* = .014, adjusted odds ratio 16.53, 95% CI 1.78–153.44).

**Conclusion:**

Preoperative ESBL-producing *E. coli* colonization was common among patients undergoing orthopedic surgery and possibly increased risk of SSIs. Further studies for multidrug-resistant GNB screening, surgical outcomes, and antibiotic prophylaxis modification should be considered in endemic regions.

## Introduction

Infections caused by multidrug-resistant organisms (MDROs) have been increased substantially worldwide and represent a major threat to the effectiveness of antimicrobial therapy and patient safety.^1–4^ MDROs are generally defined as bacteria that exhibit resistance to several antimicrobial agents, including methicillin-resistant *Staphylococcus aureus* (MRSA), vancomycin-resistant enterococci (VRE), extended-spectrum *β*-lactamase (ESBL)-producing Gram-negative bacteria (GNB), carbapenem-resistant *Enterobacterales* (CRE), carbapenem-resistant *Acinetobacter baumannii* (CRAB), and carbapenem-resistant *Pseudomonas aeruginosa* (CRPA).^3^ These pathogens have been associated with healthcare-associated infections (HAIs).^2–9^ In Southeast Asia, CRAB has emerged as one of the most prevalent pathogens associated with nosocomial infections, followed by CRPA.^10–12^ In Thailand, the high prevalence of ESBL-producing GNB colonization (29–63%) have been a public health concern and associated with inappropriate antimicrobial use.^11–15^ Furthermore, community-acquired infections caused by ESBL-producing GNB have been reported and are associated with adverse clinical outcomes.^11,15,16^ This problem has raised a concern over the MDROs colonization in healthy patients undergoing elective surgery.^10–13,16^

Orthopedic procedures are traditionally classified as clean surgeries with relatively low rates of postoperative infection. Data from the United States Centers for Disease Control and Prevention’s National Healthcare Safety Network (CDC’s NHSN) reported surgical site infections (SSIs) rates following total knee replacement ranging from 0.68% to 1.60% and from 0.67% to 2.4% following hip replacement between 2006 and 2008.^17^ Importantly, orthopedic SSIs have been associated with substantial morbidity, including prolonged hospitalization, increased readmission rates, and higher healthcare costs. Previous studies have been reported a high prevalence of MDROs isolated from SSIs, with ESBL-producing organisms and MRSA.^18,19^

Despite the growing burden of MDRO colonizations and its potential postoperaive clinical implications, the prevalence and clinical impaction of MDROs colonization among patients undergoing orthopedic surgery in Thailand remain limited. Therefore, we conducted a prospective study to evaluate the incidence, risk factors, and clinical outcomes associated with MDRO colonizations in patients undergoing elective orthopedic surgery.

## Methods

### Study setting and participants

Thammasat University Hospital (TUH) is a 650-bed tertiary-care university hospital located in the central part of Thailand. The Orthopedic Surgery service comprises a 60-bed inpatient unit staffed by 17 full-time orthopedic surgeons and 24 trainees. We conducted a prospective surveillance study of MDROs colonization among adult patients older than 18 years, undergoing elective orthopedic surgery at TUH between March and August 2016. The participants had no sign of acute infection and had not received systemic antibiotic treatment within 7 days prior to the screening. The study protocol was approved by the Ethical Committee No. 1, Faculty of Medicine, Thammasat University. All participants provided signed informed consent before the screening process.

### Microbiological methods

Surveillance swabs (BD CultureSwab™, Becton Dickinson Diagnostics, Sparks, MD, USA) were collected from 4 anatomical sites (anterior nares, throat, groin, and rectum) upon admission to the orthopedic unit. Each specimen was processed immediately and inoculated into tryptic soy broth containing a 30-μg ceftriaxone disk (bioMérieux, France), followed by incubation at 37°C. Within 48 hours of incubation, 100-μL aliquots of broth demonstrating visible turbidity were subcultured on to a sheep blood agar, and MacConkey agar with a 30-μg ceftriaxone disk and incubated at 37°C overnight. All recovered isolates within the inhibition zones were subjected to routine identification using the Vitek®2 automated system (bioMérieux, France). Antimicrobial susceptibility testing was performed according to Clinical and Laboratory Standards Institute (CLSI) interpretive criteria using the disk diffusion method and further confirmed by ESBL double-disk synergy testing for *Enterobacterales.* ^20^ MDROs were defined as MRSA, VRE, ESBL-producing GNB, CRE, CRAB, CRPA, and non-glucose fermenting GNB resistant to at least 3 antimicrobial classes.

### Data collection and statistical analysis

The primary outcome was the incidence of MDROs colonization upon hospital admission. Clinical characteristics, risk factors for MDROs colonization and infection, surgical procedures, perioperative antibiotic prophylaxis, and clinical outcomes were reviewed from electronic medical records. Clinical outcomes included operative time, estimated intraoperative blood loss, duration of hospital stay, re-hospitalization, SSIs and postoperative complications at 1, 3, 6, and 12 months following surgery. SSIs were defined according to the criteria of the CDC’s NHSN guideline for infections involving the skin, subcutaneous tissue, or deep soft tissues of the incision, or organs/spaces manipulated during an operative procedure, occurring within 30 days postoperatively or within one year in cases involving prosthetic implantation.^17^ Comparative analyses were performed between patients with and without MDROs colonization, and between patients with and without SSIs. Statistical analyses were conducted using SPSS software (version 15.0; SPSS Inc., Chicago, IL, USA). Categorical data was compared using the χ^2^ test or Fisher’s exact test, as appropriate, while continuous data was analyzed using the Student *T*-test.

## Results

### Patients’ clinical characteristics and incidence of MDROs colonization

A total of 384 surveillance swabs were collected from 96 patients (median age, 58 years). ESBL-producing *Escherichia coli* was identified in 38 swabs from 31 patients, corresponding to a colonization rate of 32.3%. There were no additional types of MDROs detected in surveillance cultures. Only one patient had a history of hospital admission within the previous year. The common procedures were involved prosthetic implantation (74 patients, 77.1%). Twenty-eight patients (30.2%) underwent total knee arthroplasty (TKA), and 15 patients (15.6%) underwent hip surgery. Perioperative antibiotic prophylaxis included cefazolin in 84 patients (87.5%), vancomycin in 8 patients (8.3%), and clindamycin in 4 patients (4.2%) (Table 1).

**Table 1.** Characteristics of 96 patients undergoing elective orthopedic surgery

Variable	No. (%)
Age, median year (IQR)	58 (44–67)
Male	31 (32.3)
Diabetes	22 (22.9)
History of systemic steroids/immunosuppressive drugs use	7 (7.3)
Rheumatoid arthritis, gout	9 (9.4)
History of MDROs infection	0
Antibiotic treatment in the past year	0
Admission in the past year	1 (1.0)
**Procedure**	
Total knee arthroplasty	28 (29.2)
Hip	15 (15.6)
Upper extremities (other than shoulder)^a^	13 (13.5)
Lower extremities (other than hip and knee)^b^	11 (11.5)
Spine	11 (11.5)
Knee (other than TKA)^c^	10 (10.4)
Shoulder^d^	6 (6.3)
Arthroscopic related procedures (any site)^e^	12 (12.5)
Prosthesis related procedures (any site)^f^	74 (77.1)
**Perioperative antibiotic prophylaxis**	
Cefazolin	84 (87.5)
Vancomycin	8 (8.3)
Clindamycin	4 (4.2)
**MDROs screening**	
ESBL-producing *E. coli* colonization	31^g^ (32.3)
**Outcomes**	
Duration of the operation, median hour (IQR)	2.45 (2.20–3.28)
Estimated blood loss from procedures, median mL. (IQR)	50 (20–250)
Total length of hospital stay, median day (IQR)	3 (2–4)
Surgical site infections	7^h^ (7.3)
Re-hospitalization during 6 months after operation	3 (3.1)

Note. No, number of patients; MDROs, multidrug-resistant organisms; ESBL, extended-spectrum betalactamase; IQR, inter quartile range; total knee arthroplasty, TKA; surgical site infections, SSIs; GNB, Gram-negative bacteria.

^a^Surgery related to elbow (n = 2), ulnar (n = 7) and hand (n = 4).

^b^Surgery related to ankle (n = 2), foot (n = 5) and arthroscopic ankle (n = 3).

^c^Surgery related to knee, anterior cruciate ligament reconstruction (ACLR) (n =5) and arthroscopic ACLR (n=6).

^d^Surgery related to clavicle (n = 2), shoulder (n = 2), and arthroscopic shoulder surgery (n = 2).

^e^Arthroscopic surgery related to knee (n = 6), elbow (n = 1), shoulder (n = 2), and ankle (n = 3).

^f^Prosthetic surgery related to knee (TKA) (n = 28), and hip arthroplasty (n = 14).

^g^38 ESBL-producing *E. coli* isolates (7 groin swabs, 31 rectal swabs) recovered from 31 patients.

^h^7 patients with SSIs related to TKA (n = 3), spine surgery with prosthetic implementation (n = 2), hip surgery (n = 1) and foot surgery (n = 1).

### MDRO isolates and in-vitro antimicrobial susceptibility testing

Among the 38 ESBL-producing *E. coli* isolates, 31 were collected from the rectal area and 7 from the groin area. Total of 31 ESBL-producing *E. coli* isolates from individual patients were highly in-vitro susceptible to carbapenems (100%), piperacillin/tazobactam (100%), amikacin (100%) and cefoperazone/sulbactam (80.2%) but were poorly susceptible to gentamicin (38.5%), trimethoprim–sulfamethoxazole (46.2%), norfloxacin (65.4%), and ciprofloxacin (61.5%). All ESBL-producing *E. coli* isolates were confirmed by a positive ESBL double-disk synergy test and the highly in-vitro susceptible to cefoxitin (88.5%) (Table 2).

**Table 2.** In-vitro antimicrobial susceptibility pattern of 31 ESBL-producing *E. coli* isolates recovered from individual patients undergoing elective orthopedic surgery

Antibiotics	Number of isolates with antibiotic susceptible (%)
Meropenem	31 (100)
Imipenem/cilastatin	31 (100)
Ertapenem	31 (100)
Doripenem	31 (100)
Cefazolin	0 (0)
Cefoxitin	27 (88.5)
Ceftriaxone	1 (3.8)
Ceftazidime	13 (42.3)
Cefpirome	1 (3.8)
Amoxicillin/clavulanic acid	17 (53.8)
Piperacillin/tazobactam	31 (100)
Cefoperazone/sulbactam	25 (80.8)
Trimethoprim/sulfamethoxazole	14 (46.2)
Gentamicin	12 (38.5)
Amikacin	31 (100)
Norfloxacin	20 (65.4)
Ciprofloxacin	19 (61.5)

*Note*. ESBL, extended-spectrum betalactamase. All ESBL-producing *E. coli* isolates were confirmed with positive ESBL double-disk test.

### MDROs colonization, risk factors, and outcomes

Patients were followed for 12 months. The clinical outcomes were monitored. Seven patients (7.3%) developed SSIs during the follow-up period. The rate of SSIs was higher among patients colonized with ESBL-producing *E. coli* compared with those without colonization (6/31, 19.4% vs. 1/65, 1.5%; *P* = .004; odds ratio [OR] 15.36; 95% confidence interval [CI], 1.67–356.32). There were no differences in other clinical characteristics, procedure types, and postoperative outcomes between patients with and *without MDROs colonization* (Table 3).

**Table 3.** Characteristics and outcomes of patients with and without ESBL-producing *E. coli* colonization

Variable	ESBL-producing *E. coli* colonization (n = 31) No. (%)	No ESBL-producing *E. coli* colonization (n = 65) No. (%)	Odds Ratio	95% CI	*P*-value
Age, median year (IQR)	59 (37–66)	57 (47–68)	–	–	0.636
Male	13 (42)	18 (27.7)	1.89	0.70–5.10	0.171
Diabetes	8 (25.8)	14 (21.5)	1.20	0.50–2.70	0.796
History of steroids/immunosuppressive drugs	2 (6.5)	5 (7.7)	0.83	0.10–5.27	1
Rheumatoid arthritis, gout	3 (9.7)	5(7.7)	1.29	0.22–6.82	0.710
History of MDROs infection	0	0	–	–	1
Antibiotic treatment in the past year	0	0	–	–	1
Admission in the past year	0	1(1.5)	–	1	1
**Procedure**					
Total knee arthroplasty	7 (22.6)	21 (32.3)	0.61	0.20–1.81	0.472
Hip	6 (19.4)	9 (13.8)	1.49	0.42–5.27	0.552
Upper extremities (other than shoulder)	3 (9.7)	10 (15.4)	0.59	0.12–2.61	0.538
Lower extremities (other than hip and knee)	1 (3.3)	7 (10.8)	0.28	0.01–2.43	0.430
Spine	4 (12.9)	7 (10.8)	1.23	0.27–5.25	0.743
Knee (other than TKA)	5 (16.1)	5 (7.7)	2.31	0.52–10.29	0.284
Shoulder	3 (9.7)	3 (4.6)	2.21	0.33–14.94	0.384
Arthroscopic related procedures (any site)	5 (16.1)	7(10.8)	1.59	0.39–6.34	0.516
Prosthesis related procedures (any site)	27(87.1)	47(72.3)	2.59	0.72–10.13	0.126
**Perioperative antibiotic prophylaxis**					
Cefazolin	25 (80.6)	79 (88.8)	0.32	0.04–2.73	0.211
Vancomycin	3 (9.7)	6 (6.7)	5.53	0.59–45.46	0.104
Clindamycin	3 (9.7)	3 (3.4)	4.78	0.17–69.87	0.265
**Outcomes**					
Duration of the operation, median hour (IQR)	2.5 (2.25–3.40)	2.45 (2.13–3.18)	–	–	0.375
Estimated blood loss from procedures, median mL. (IQR)	50 (20–150)	50 (20–250)	–	–	0.296
Total length of hospital stay, median day (IQR)	3 (2–5)	3 (2–4)	–	–	0.268
Surgical site infections	6 (19.4)	1 (1.5)	15.36	1.67–356.32	**0.004**
Re-hospitalization during 6 months after operation	2 (6.5)	1 (1.5)	4.4	0.30–128.55	0.243

*Note*. No, number of patients; MDROs, multidrug-resistant organisms; ESBL, extended-spectrum betalactamase; IQR, inter quartile range; total knee arthroplasty, TKA; surgical site infections, SSIs; GNB, Gram-negative bacteria.

### Patients with surgical site infections

Seven patients developed SSIs following TKA (n = 3), spine surgery with prosthetic implantation (n = 2), hip surgery (n = 1), and foot surgery (n = 1). Six of these patients (85.7%) were colonized with ESBL-*E. coli* preoperatively. Moreover, ESBL-producing *E. coli* colonization and re-hospitalization were significantly higher among patients with SSIs compared with those without SSIs. From the multivariate logistic regression analysis, preoperative ESBL-producing *E. coli* colonization was associated with the development of SSIs (*P* = .014; adjusted OR, 16.53; 95% CI 1.78–153.44) (Table 4).

**Table 4.** Characteristics of patients with and without surgical site infections

Variable	SSIs (n=7) No. (%)	No SSIs (n= 89) No. (%)	Odds Ratio	95% CI	*P*-value
Age, median year (IQR)	62 (51–65)	57 (42–68)	–	–	0.967
Male	2 (28.6)	29 (32.6)	0.83	0.10–5.27	1
Diabetes	1 (14.3)	21 (23.6)	0.54	0.02–5.03	0.58
History of systemic steroids/immunosuppressive drugs	0	7(7.9)	0	0–11.61	1
Rheumatoid arthritis, gout	1 (14.3)	8 (9.0)	1.69	0.07–18.07	0.51
History of MDROs infection	0	0	0	0	1
Admission in the past year	0	1 (1.1)	0	0–252.20	1
Antibiotic treatment in the past year	0	0	0	0	1
**Procedure**					
Total knee arthroplasty	3 (42.9)	25 (28.1)	1.92	0.31–11.23	0.41
Knee (other than TKA)	0	10 (11.2)	0	0–7.38	1
Spine	2 (28.6)	9 (10.1)	3.56	0.41–26.23	0.182
Hip	1 (14.3)	14 (15.7)	0.89	0.04–8.68	1
Shoulder	0	6 (6.7)	0	0–14.15	1
Upper extremities (other than shoulder)	0	13 (14.6)	0	0–5.28	0.59
Lower extremities (other than hip and knee)	1 (14.3)	10 (11.2)	1.32	0.05–13.48	0.586
Arthroscopic related procedures (any site)	0	12 (13.5)	0	0–5.85	0.59
Prosthesis related procedures (any site)	5 (71.4)	69 (77.5)	0.73	0.11–5.88	0.66
**Antibiotic prophylaxis**					
Cefazolin	5 (71.4)	79 (88.8)	0.32	0.04–2.73	0.211
Vancomycin	2 (28.6)	6 (6.7)	5.53	0.59–45.46	0.104
Clindamycin	1 (14.3)	3 (3.4)	4.78	0.17–69.87	0.265
Duration of the operation, median hour (IQR)	3 (2.25–3.3)	2.45 (2.18–3.25)	–	–	0.7
Estimated blood loss from procedures, median ml. (IQR)	150 (30–300)	50 (20–250)	–	–	0.841
Total length of stay, median day (IQR)	3 (2–8)	3 (2–4)	–	–	0.531
ESBL-producing *E. coli* colonization	6 (85.7)	25 (28.1)	15.36	1.67–356.32	**0.004**
Re-hospitalization during 6 months after operation	2 (28.6)	1 (1.1)	35.20	1.95–1207.44	**0.013**

*Note*. No, number of patients; SSIs, surgical site infections; MDROs, multidrug-resistant organisms; ESBL, extended-spectrum betalactamase; IQR, inter quartile range.

From the multivariate logistic regression analysis (ESBL-producing *E. coli* colonization, and re-hospitalization), ESBL*-E.coli* colonization was associated with SSIs (*P*=0.014, adjusted odds ratio 16.53, 95% CI 1.78–153.44).

All patients with SSIs were initially treated with empirical oral antibiotics (dicloxacillin or amoxycillin/clavulanate) and outpatient management. Four patients demonstrated clinical improvement following oral antibiotic treatment. Three patients with SSIs experienced clinical treatment failure following initial oral antibiotic treatment and required re-hospitalization. Surgical incision and drainage were subsequently performed with intravenous broad-spectrum antibiotic treatment (piperacillin/tazobactam or meropenem). However, microbiological confirmation of SSIs was unavailable as the surgical wound cultures yielded no growth.

## Discussion

We observed a high incidence of preoperative ESBL-producing *E. coli* colonization (32.3%) and a high rate of SSIs (7.3%) among patients undergoing elective orthopedic procedures. Preoperative ESBL-producing *E. coli* colonization was independently associated with the development of SSIs. These findings highlighted the potential clinical relevance of MDROs colonization in surgical populations in endemic regions.^14,15^ In our study, only one patient had a history of hospital admission within the prior year, suggesting that traditional healthcare-associated risk factors may not adequately capture the current epidemiology of MDROs colonization. In the settings with a high prevalence of antimicrobial resistance, community-acquired colonization with MDROs might be an important reservoir for subsequent infection. Increasing reports of community-based ESBL-producing GNB colonization among healthy individuals in endemic regions support the hypothesis that asymptomatic colonization may influence perioperative infectious risk, even in patients without established healthcare exposures.^16,19,22–25^

A previous meta-analysis by Karanika *et al.* reported a global prevalence of fecal ESBL-producing GNB colonization of approximately 14% among healthy adults, with substantially higher rates in parts of the West Pacific and Southeast Asia, Africa, and the eastern Mediterranean (22%–46%).^16^ The colonization rate observed in our study was consistent with these regional estimates. Colonization with MDROs especially in multidrug-resistant GNB have been associated with subsequent infection in several clinical settings.^26–31^ Data regarding the clinical impact of gastrointestinal colonization with multidrug-resistant GNB on postoperative infectious outcomes remain limited, particularly in patients undergoing elective surgical procedures.^28–30^ However, few studies have been reported the risk for fecal multidrug-resistant GNB colonization and infection, particularly in patients with underlying hematologic malignancies.^28,29^ Cheikh *et al*. performed a retrospective cohort study of pediatric cardiac surgery in Morocco. ESBL-producing GNB colonization was around 15% (17/111). Among the 17 patients, 23.5% (4/17) developed a postoperative infection due to ESBL-producing GNB.^30^ In Thailand, preoperative screening for ESBL-producing GNB colonization was conducted in patients underwent abdominal surgery. ESBL-producing GNB colonization and dirty wound classification were associated with SSIs. However, there was no association between carbapenem prophylaxis and reduction in SSIs.^31^

Our study is the first study to assess the incidence of SSIs after elective orthopedic procedures in Thailand. We found a high rate of SSIs compared to CDC’s NHSH report (<3%).^21^ Our study supports the data from the International Nosocomial Infection Control Consortium (INICC) (resource-limited settings) that there are higher rates of SSIs postsurgery. The data from INICC has been collected from a multinational, multicenter, collaborative on health care associated control program that used a surveillance system. This system is in 30 countries in Latin America, Asia, Africa, and Europe which are MDROs endemic regions. For example, the rate of SSIs after hip prosthesis was 2.6% (INICC data) versus 1.3% (CDC’s NHSN data).^21^ Elective orthopedic procedures are considered as a clean procedure with low rate of SSIs. The rates of SSIs were higher in patients with certain risk factors based on the patient’s underlying disease, immune status, and procedure types.^17^ High rate of SSIs in MDROs endemic region may be related to the virulence of the pathogen, antimicrobial resistance and lack of infection prevention and control program.

In our study, the association between ESBL-producing *E. coli* colonization and development of SSIs was consistent with previous studies.^30,31^ However, we had only 7 patients with SSIs and limited microbiological culture confirmation. Three patients with SSIs who had re-hospitalization were response with empirical of board spectrum antibiotic treatment. Thus, we had insufficient evidence to clarify the process of SSIs development. Nevertheless, ESBL-producing GNB colonization may be enhanced or be the causative pathogen by hematological route from gut translocation, or direct contamination at the skin surface at surgical sites. Active surveillance screening of ESBL-producing GNB colonization for patients undergoing elective orthopedic surgery may have a significant contribution on the antibiotic perioperative prophylaxis and infection control implementation. However, the cost-effective analysis of the active surveillance screening and outcomes required further studies.

In our study, MDROs surveillance cultures recovered only ESBL-producing *E. coli*. CREs, CRAB, CRPA, MRSA, and VREs isolates were not recovered. ESBL-producing *E. coli* is the major community associated MDROs pathogen in Thailand which is consistent with previous studies.^11,13–15,21,32^ Although prevalence of CREs infections has been increasing in Thailand. It may be possible that it is still related to nosocomial pathogen.^10,11,32^ The recent national surveillance molecular characterization of multidrug-resistant GNB from the Research University Network Thailand study reported the high prevalence of extended-spectrum cephalosporinase *E. coli* (14.5%) and the low prevalence of carbapenem-resistant *E.coli* (1.3%).^32^ In our study, the patients with elective orthopedic surgery had no traditional risk of MDROs. Thus, the positive ESBL-*E. coli* colonization could be reflected the ESBL-producing *E. coli* colonization in the community setting which were higher than CRE colonization.

The global strains of *E. coli* ST 131, that carry the CTX-M gene may be the major strain of ESBL-producing *E. coli* isolate from our study. From our study, all ESBL-producing *E. coli* strains were highly susceptible to carbapenems. However, the control of carbapenems use should be considered. Thus, piperacillin/ tazobactam (in noncritical infections), amikacin, and Cefoperazone/sulbactam are considered as alternative regimen. The rectal swab culture revealed a great performance for detecting ESBL-producing *E. coli* colonization. Our study was also confirmed the low prevalence of community-acquired MRSA in Thailand. Active surveillance screening for MRSA in patients without risk factors for HAIs and anti-MRSA perioperative prophylaxis are not recommended.

### Limitation

This study has several limitations. First, it was conducted at a single center, which may limit generalizability. Second, molecular characterization of ESBL-producing *E. coli* isolates was not performed. Third, we had a small number of patients with SSIs, which may limit microbiological confirmation and the interpretation of the association between colonization and postoperative infection risks. The clinical significance of ESBL-producing *E. coli* colonization, clinical outcomes, and antimicrobial perioperative prophylaxis adjustment in orthopedic patients require further investigation.

### Conclusion

Preoperative ESBL-producing *E. coli* colonization was common among patients undergoing elective orthopedic surgery in the endemic setting and was associated with an increased risk of SSIs. These findings suggest that colonization may serve as a potential marker of postoperative infectious risk. Further studies are needed to evaluate the role of targeted surveillance and perioperative antibiotic prophylaxis modification in patients colonized with MDROs particularly in GNB.
